# Do Longer Exhalations Increase HRV During Slow-Paced Breathing?

**DOI:** 10.1007/s10484-024-09637-2

**Published:** 2024-03-20

**Authors:** Zachary M. Meehan, Fred Shaffer

**Affiliations:** 1 Department of Psychological and Brain Sciences, University of Delaware, 105 The Green, Wolf Hall, Newark, DE 19716 USA; 2 Center for Applied Psychophysiology, Truman State University, Kirksville, MO USA

**Keywords:** Slow-paced breathing, Inhalation-to-exhalation ratio, Respiratory sinus arrhythmia, Heart rate variability

## Abstract

Slow-paced breathing at an individual’s resonance frequency (RF) is a common element of heart rate variability (HRV) biofeedback training (Laborde et al. in Psychophysiology 59:e13952, 2022). Although there is strong empirical support for teaching clients to slow their respiration rate (RR) to the adult RF range between 4.5 and 6.5 bpm (Lehrer & Gevirtz, 2014), there have been no definitive findings regarding the best inhalation-to-exhalation (IE) ratio to increase HRV when breathing within this range. Three methodological challenges have frustrated previous studies: ensuring participants breathed at the target RR, IE ratio, and the same RR during each IE ratio. The reviewed studies disagreed regarding the effect of IE ratios. Three studies found no IE ratio effect (Cappo & Holmes in J Psychosom Res 28:265-273, 1984; Edmonds et al. in Biofeedback 37:141-146, 2009; Klintworth et al. in Physiol Meas 33:1717-1731, 2012). One reported an advantage for equal inhalations and exhalations (Lin et al. in Int J Psychophysiol 91:206?211, 2014). Four studies observed an advantage for longer exhalations than inhalations (Bae et al. in Psychophysiology 58:e13905, 2021; Laborde et al. in Sustainability 13:7775, 2021; Strauss-Blasche et al. in Clin Exp Pharmacol Physiol 27:601?60, 2000; Van Diest et al. in Appl Psychophysiol Biofeedback 39:171?180, 2014). One study reported an advantage for longer inhalations than exhalations (Paprika et al. in Acta Physiol Hung 101:273?281, 2014). We conducted original (*N* = 26) and replication (*N* = 16) studies to determine whether a 1:2 IE ratio produces different HRV time-domain, frequency-domain, or nonlinear metrics than a 1:1 ratio when breathing at 6 bpm. Our original study found that IE ratio did not affect HRV time- and frequency-domain metrics. The replication study confirmed these results and found no effect on HRV nonlinear measurements.

## Introduction

There is strong theoretical and empirical support for using resonance frequency breathing to maximize heart rate variability (HRV). Specifically, the *resonance frequency* (RF) is the stimulation rate that produces the largest heart rate (HR) oscillations (Lehrer & Eddie, 2013; van de Vooren et al., 2007; Vaschillo et al., 2002, 2006, 2011). Indeed, gold standard manuals for HRV biofeedback teach clients to slow their respiration rate to the RF range, which is between 4.5 and 6.5 bpm for most adults (Lehrer & Gevirtz, 2014; Lehrer et al., 2000; Shaffer & Meehan, 2020; Vaschillo et al., 2002). Breathing in this range optimizes respiratory sinus arrhythmia (RSA), or the speeding and slowing of HR across the breathing cycle. This pattern results from the baroreflex system’s closed negative-feedback loops regulating HR and blood pressure. The increased magnitude of HR oscillations is due to the HR baroreflex closed loop’s intrinsic resonance properties (Lehrer, 2022).

The extant literature disagrees regarding the best inhalation-to-exhalation (IE) ratio when breathing within the RF range. In theory, a 1:2 ratio would increase cardiac vagal activity more than a 1:1 ratio if extended exhalation prolongs the parasympathetic slowing of the heart (Laborde et al., 2022). In other words, breathing at a 1:2 ratio should theoretically produce greater RSA as measured by time- and frequency-domain metrics. However, the disparate findings make consensus challenging. Specifically, in nine studies, researchers came to four different conclusions regarding the effects of breathing at various IE ratios (Bae et al., 2021; Cappo & Holmes, 1984; Edmonds et al., 2009; Klintworth et al., 2012; Laborde et al., 2021; Lin et al., 2014; Paprika et al., 2014; Strauss-Blasche et al., 2000; Van Diest et al., 2014). However, these studies present methodological challenges that may have influenced the findings.

## Methodological Challenges

Disparate findings may be due to several reasons. For one, unmeasured moderators may account for variability in outcomes across samples. For example, researchers who fail to replicate outcomes from a given treatment may rely on samples with distinct characteristics (e.g., older vs. younger participants) that influence the association between the independent and dependent variables (Kazdin, 2016). However, a common reason is differences in the methodological rigor of studies, which can impact their internal and external validity. Confounding variables, specifically, are third variables that may influence both the dependent and independent variables. Poor internal validity, or low control over these potential confounding variables, may lead to spurious and often contradictory outcomes. As such, we sought to rule out potential confounding variables by first examining the methodological challenges of conducting research in IE ratios and then conducting a replication that more tightly controls these variables.

### Ensuring Internal Validity

The uniqueness of the RF training exercises warrants special attention to address three potential challenges to delivering the intervention. First, researchers must confirm that participants breathed at the target rate. If participants breathed faster than 6.5 bpm, the findings might not apply to RF breathing, as RF breathing refers to the 4.5–6.5 bpm (Lehrer & Gevirtz, 2014). Second, researchers must ensure that participants breathed at the assigned IE ratios to manipulate the independent variable successfully. If the researchers assigned a participant to breathe at a 1:2 ratio, but the participant achieved a 3:7 ratio, then the conclusions regarding a 1:2 ratio would be spurious. Third, researchers must ensure that participants breathed at the same frequency, or respiration rate (RR), in each IE condition. If the group trained for a 1:1 ratio breathed at 5.5 bpm, and the group trained at a 1:2 ratio breathed at 6.5 bpm, the findings would be confounded by respiration rate.

### Ensuring Statistical Power

Power refers to the likelihood of detecting an effect if one exists (Kazdin, 2016). Researchers should ideally perform a power analysis before recruiting participants. They should always report on the power of their experimental analyses. Power of 80% or higher is considered acceptable (Bezeau & Graves, 2001). Ioannidis (2005) considers power critical, as insufficient power can result in inflated Type 1 (false positives) and Type 2 errors (false negatives). Because power depends on several study factors (e.g., sample size, alpha level, anticipated effect size), there is no universal solution. Rather, researchers must closely examine these factors when designing, implementing, and reporting experiments.

## Literature Review

We conducted a review to summarize the extant literature on the effects of IE ratio in humans. Following this review, we used the revised Cochrane risk-of-bias tool for randomized trials to assess different aspects of trial design, conduct, and reporting (Higgins et al., 2011). This tool guides reviewers in a standardized format to track five potential sources of bias: (a) randomization process, (b) delivery of the intervention, (c) handling of missing data, (d) measurement of the outcome(s), and (e) selection of the reported results. The following summaries evaluate these factors while describing their methods and results.

### Cappo and Holmes

Cappo and Holmes (1984) investigated 60 undergraduates in a between-subjects design. Physiological monitoring used a finger pulse transducer and an impedance pneumograph. They randomly assigned participants to breathe at fast-slow (1:4), slow-fast (4:1), or equal (1:1) ratios. However, the authors did not test baseline differences across the groups. Blood pressure and HR did not differ across the breathing ratio conditions. The authors did not measure HRV. There also appear to be no concerns related to missing data. Due to their technology’s imprecision, judges reviewed the respiration records to confirm compliance with the breathing ratio instructions. However, we cannot know how closely participants adhered to each condition’s IE ratio. In conclusion, this study is at high risk of bias due to the lack of tests for baseline differences, lack of manipulation checks for the IE ratios, and failure to use HRV metrics when making conclusions regarding physiological arousal.

### Strauss-Blasche and Colleagues

Strauss-Blasche and colleagues (2000) assigned 12 healthy participants to 2-minute, controlled 6-bpm breathing trials in which they inhaled slowly and exhaled quickly (LISE) or the opposite pattern (SILE). In this cross-over design, the investigators instructed half of their participants in the LISE or SILE pattern for 30 min. The researchers monitored HRV using an ECG and respiration with strain gauges around the abdomen and thorax. They measured pulse volume amplitude using a PPG sensor placed on the fourth finger of the non-dominant hand. The investigators measured RR and IE ratios during the two ratio conditions. RR was 9.6 ± 3.1 in the LISE condition compared with 10.0 ± 3.1 in the SILE condition—there was no significant difference. Both scores fell *abov*e the adult RF range between 4.5 and 6.5 bpm (Lehrer & Gevirtz, 2014). Their participants did not follow the LISE instructions because their IE ratio was 1.0 ± 0.3. They followed the SILE instructions with an IE ratio of 3.4 ± 0.8. Finally, HR, log RSA, and high-frequency (HF) power were higher in the SILE condition, whereas pulse volume amplitude was higher in the LISE condition. In conclusion, this study is at high risk of bias due to a lack of counterbalancing of conditions and participant failure to breathe at the intended RR or IE ratios.

### Edmonds and Colleagues

Edmonds and colleagues (2009) randomized 14 healthy adult participants to different orders of four 6-bpm IE ratios and a fifth condition in which they breathed in phase with the HR waveform. Following a 5-min baseline, participants completed four 5-min IE conditions, separated by 2-min rest periods. These included C1–1:1 ratio with 1.25-s sustain and pause; C2–1:1 ratio with a 10-ms sustain and pause; C3–1:2 ratio with no sustain or pause; and 1:2 ratio with a 1.25-s sustain and pause. The authors did not report their participants’ actual RR or success following the breathing instructions. They did not report inferential tests comparing breathing condition HRV statistics. The pNN50, SDNN, percentage of high-frequency power (HF%), percentage of low-frequency power (LF%), and percentage of very-low-frequency power (VLF%) values for each condition fell within the margin of error. There is insufficient information to evaluate the risk of bias, as the authors did not report participants’ RR, success following instructions, or the inferential tests comparing HRV statistics across breathing conditions.

### Klintworth and Colleagues

Klintworth and colleagues (2012) assigned 18 healthy volunteers to breathe for 6 min each at a 1:2, 1:1, and 2:1 ratio while supine. The authors monitored ECG with three surface electrodes and breathing with a thermistor under the nose. The researchers stabilized the participants in a supine position for 15 min. The researchers trained the participants to breathe during 4.5-s cycles guided by a metronome during the adaptation period. The five trials were presented in a fixed order, consisting of 1- controlled inhalation, voluntary exhalation; 2 – inhalation and exhalation controlled at a 1:2 ratio; 3 - inhalation and exhalation controlled at a 1:1 ratio; 4 - inhalation and exhalation controlled at a 2:1 ratio; and 5 - controlled inhalation, voluntary exhalation. The authors did not report the actual RRs or IE ratios. The thermistor breathing pattern was visually inspected for compliance with IE ratio instructions. Although breathing pattern affected heart rate asymmetry (HRA), it did not affect the RMSSD, the SDNN, normalized LF power (LFnu), normalized HF power (HFnu), the LF/HF, and SD1. In sum, this study is at high risk of bias due to the potential for order effects and no reporting of achieved RRs or IE ratios.

### Lin and Colleagues

Lin and colleagues (2014) randomly assigned 47 healthy undergraduates to breathe at 5.5 or 6 bpm with 5:5 or 4:6 ratios in four 3-min conditions guided by a video pacing display. The researchers measured HR, HRV, and breathing using an ECG and respirometer. They used Latin square counterbalancing to control order effects. After a 5-min baseline, participants performed one of four sequences. They breathed at each assigned rate/ratio for 2 min, followed by a 1-min buffer. They concluded with a 5-min recovery period. The authors did not confirm their participants’ actual RR and IE ratio in each condition. Their Fisher’s LSD post hoc analysis confounded RR with IE ratio for the SDNN, comparing 5.5 bpm (5:5) with 6 bpm (4:6). They directly compared 5.5 bpm (5:5) with 5.5 bpm (4:6). LF power was higher when breathing at a 5:5 ratio. Finally, they directly compared 6 bpm (5:5) with 6 bpm (4:6). The LF/HF ratio was higher when breathing at a 5:5 ratio. In conclusion, this study is at high risk of bias due to a lack of reporting on achieved RRs or IE ratios and confounding RR with IE ratio when analyzing SDNN.

### Paprika and Colleagues

Paprika and colleagues (2014) studied 24 volunteers using ECG electrodes during three breathing sessions. Participants completed a 15-min acclimation period, followed by a 6-min resting baseline in the supine position. The researchers instructed participants to follow verbal commands for inspiration and expiration. All participants first completed symmetrical breathing exercises (5:5 IE ratio), followed by a 3:7 and 7:3 IE ratio. The authors reported that the order was randomly assigned but did not report the randomization method. The authors did not report manipulation checks to determine the respiration rate or whether participants breathed at the instructed rates. The authors reported PNN50, RMSSD, and LF power changes that favored the longer inhalation period (7:3 IE ratio). In sum, this study is at high risk of bias due to the potential for order effects and no reporting of achieved RRs or IE ratios.

### Van Diest and Colleagues

Van Diest and colleagues (2014) studied 23 undergraduates in a within-subjects design, monitoring ECG and respiration for different RR and IE ratio conditions. Following instruction, an experimenter supervised the practice of a fixed sequence of breathing rates and IE ratios for 45 s each. The conditions were 12 bpm/0.42 ratio (1.5 to 3.5 s), 12 bpm/2.33 ratio (3.5 s to 1.5 s), 6 bpm/0.42 ratio (3 to 7 s), and 6 bpm/2.33 ratio (7 s to 3 s). Next, they played 5-min videos of the same conditions to guide participant breathing while the experimenter left the room. Participants did not breathe at the target rates. For 6 bpm, the rates were 7.32 (*SD* = 1.90) for the low 0.42 ratio and 7.69 (*SD* = 2.08) for the high 2.33 ratio. The Ti/Te ratio, which compared inhalation with exhalation duration, confirmed that participants followed the IE ratio instructions for the low- but not the high-ratio conditions. For 6 bpm, the actual ratios were 0.49 (*SD* = 0.06) for the low 0.42 ratio and 1.44 (*SD* = 0.26) for the high 2.33 ratio. A low 0.42 ratio was associated with higher HR, RSA, and HF power at 6 bpm than the high 2.33 ratio. The IE ratio did not affect LF power.

The Van Diest and colleagues’ (2014) study’s monitoring of the RR rate and Ti/Te ratio (IE duration) was commendable. They measured their participants’ ability to follow the video pacing displays. However, their study was confounded since practice and treatment condition order were fixed instead of randomized. Confounding by order threatened the internal validity of their experiment. Since participants in the 6-bpm conditions breathed significantly faster than 6 bpm, their findings may not apply to 0.1 Hz or RF breathing. The finding of higher HR at 6 bpm for the 0.42 ratio is puzzling since longer exhalations should be associated with lower HR due to extended parasympathetic influence. The authors noted that this outcome was inconsistent with findings by Cappo and Holmes (1984) and speculated that intrathoracic pressure differences may have been responsible. In conclusion, this study yielded a high risk of bias because the participants did not breathe at the target rates and participants in the high-ratio condition did not follow the IE instructions.

### Bae and Colleagues

Bae and colleagues (2021) randomly assigned 28 participants to different orders of 1:1, 2:1, and 1:2 exhalation-to-inhalation (EI) ratios at spontaneous breathing rates. The researchers monitored HRV using an ECG and respiration with a pneumatic sensor around the chest. Following a 5-min resting baseline, they used the participants’ mean RR to create sound cues to guide inhalation and exhalation. They provided at least two 3-min rehearsal sessions to ensure participants could distinguish the inhalation and exhalation pitches. The participants completed three 5-min EI ratio conditions, separated by 8-min 1:1 EI ratio breathing periods. These were not true resting baselines and constituted a confound because participants received more than three times the practice in 1:1 than 2:1 or 1:2 breathing. All participants began with a 1:1 EI ratio, which confounded the breathing ratio condition with order. The researchers randomized the order of the 2:1 and 1:2 EI ratios. Since participants could not follow the 1:2 sounds, these data were excluded. The researchers reported that the actual EI ratios were 1.08 for 1:1 and 1.33 for 2:1, and their difference was significant. The RMSSD and HF power were greater in the 2:1 than 1:1 condition while breathing at 14.16 and 14.47 bpm, respectively. In conclusion, this study yielded a high risk of bias because the authors reported incomplete randomization, participants who received 1:1 IE instructions received more practice, participants did not breathe at the instructed IE ratio, and the authors did not report baseline differences.

### Laborde and Colleagues

Laborde and colleagues (2021) studied 64 athletes using ECG electrodes. They derived the RR from the ECG using a Kubios algorithm. They randomly assigned participants to six 5-min conditions separated by 5-min washout periods. The authors specified the inhalation, post-inhalation pause, expiration, and post-exhalation pauses and ratios. The six conditions included 4.6 s/0.4 s/4.6 s/0.4 s, (I/E = 1.0); 4.1 s/0.4 s/5.1 s/0.4 s, (I/E = 0.8); 5.1 s/0.4 s/4.1 s/0.4 s, (I/E = 1.2); 5 s/5 s (no pauses), (I/E = 1); 4.5 s/5.5 s (no pauses), (I/E = 0.8); 5.5 s/4.5 s (no pauses), (I/E = 1.2). The authors did not estimate baseline differences in the measured outcomes, nor did they report on manipulation checks.

The participants viewed a slow-paced breathing video during a 15-min familiarization period, followed by a 5-min resting baseline and the six breathing conditions. A video of a rising and falling ball at 6 bpm guided their breathing in each condition. Although the authors performed a manipulation check for RR, they could not confirm that participants breathed at the assigned ratios and pauses. The authors reported changes in the RMSSD as a measure of CVA since it is conceptualized as vagally mediated HRV (vmHRV; Jarczock et al., 2021). Log RMSSD was higher when exhalation was longer than inhalation and was unaffected by post-inhalation and exhalation pauses. The authors noted that their IE ratios were limited (0.8–1.2) compared to other reviewed studies. Their sample size was the largest of the reviewed studies. In conclusion, this study yielded a high risk of bias because they did not measure baseline differences or report manipulation checks.

## Research Synthesis

Our literature review summarized and critiqued studies of IE ratio effects on HR and HRV metrics during RF breathing. See Table 1 for a breakdown. The reviewed studies disagreed regarding the effect of IE ratios. Three studies (Cappo & Holmes, 1984; Edmonds et al., 2009; Klintworth et al., 2012) found no IE ratio effect. One study reported an advantage for equal inhalations and exhalations (Lin et al., 2014), and another reported an advantage for longer inhalation periods (Paprika et al., 2014). Four studies observed an advantage for longer exhalations than inhalations (Bae et al., 2021; Laborde et al., 2021; Strauss-Blasche et al., 2000; Van Diest et al., 2014). Because only Laborde and colleagues (2021) confirmed that their participants breathed at 6 bpm, this was the only study that evaluated the effect of IE ratio on HRV during RF-range breathing. Additionally, this experiment recruited athletes and reported IE effects on a single HRV metric, RMSSD; thus, we believe that studies of healthy undergraduates using a more comprehensive set of HRV metrics are needed.

**Table 1:** Characteristics of study methodology

Study	*n*	Protocol Adherence				
		RR	IE Ratio	6 bpm	Confounds	
Cappo and Holmes (1984)	60	Yes	No	No	No	
Strauss-Blasche et al. (2000)	12	Yes	Yes	No	Yes	
Edmonds et al. (2009)	14	No	No	No	No	
Klintworth et al. (2012)	18	No	No	No	Yes	
Lin et al. (2014)	47	No	No	No	Yes	
Paprika et al. (2014)	24	No	No	No	Yes	
Van Diest et al. (2014)	23	Yes	Yes	No	Yes	
Bae et al. (2021)	28	Yes	Yes	No	Yes	
Laborde et al. (2021)	64	Yes	No	Yes	No	
The Current Study – One	26	Yes	Yes	Yes	No	
The Current Study – Two	16	Yes	Yes	Yes	No	

*Note* bpm = breaths per minute; IE = inhalation-exhalation; *n* = sample size; RR = respiration rate. Reported protocol adherence refers to whether authors reported if participants followed the assigned conditions (e.g., breathing at assigned IE ratio). Confounds observed refers to whether unmeasured or uncontrolled factors may affect the measured outcome

### Current Study

We conducted original and replication studies to determine whether a 1:2 IE ratio produces different HRV time-domain, frequency-domain, or nonlinear metrics than a 1:1 ratio when breathing at 6 bpm within the RF range. *HRV time-domain metrics* quantify the variability in the interbeat interval (IBI) measurements, which is the period between successive heartbeats. *Frequency-domain measurements* quantify absolute or relative power distribution into component frequency bands. *Nonlinear measurements* quantify a time series’ unpredictability due to complex and dynamically changing control mechanisms. We did not frame a directional hypothesis due to the disagreement among the published studies.

### Hypothesis

Our original and replication studies addressed the question: Compared to a 1:1 IE ratio, does a 1:2 IE ratio increase HRV during 6-bpm SPB? Due to the disagreement among the reviewed studies, we did not make any predictions.

## Method

We are reporting on two randomized control trials (RCTs) that used identical equipment, procedures, and manipulation checks. We performed the second RCT to ensure we could replicate the first study’s provocative findings with a different undergraduate sample.

### Study One

#### Participants

Twenty-six undergraduates (10 women and 16 men), 18 to 22, participated in this study. They had one to two semesters of effortless breathing training within the adult resonance frequency range (4.5–6.5 bpm; Lehrer & Gevirtz, 2014). Extensive SPB practice enabled our participants to follow the pacing display consistently. Researchers recruited participants from the Truman Center for Applied Psychophysiology. There were no participation incentives.

#### Apparatus

**Fig. 1:**
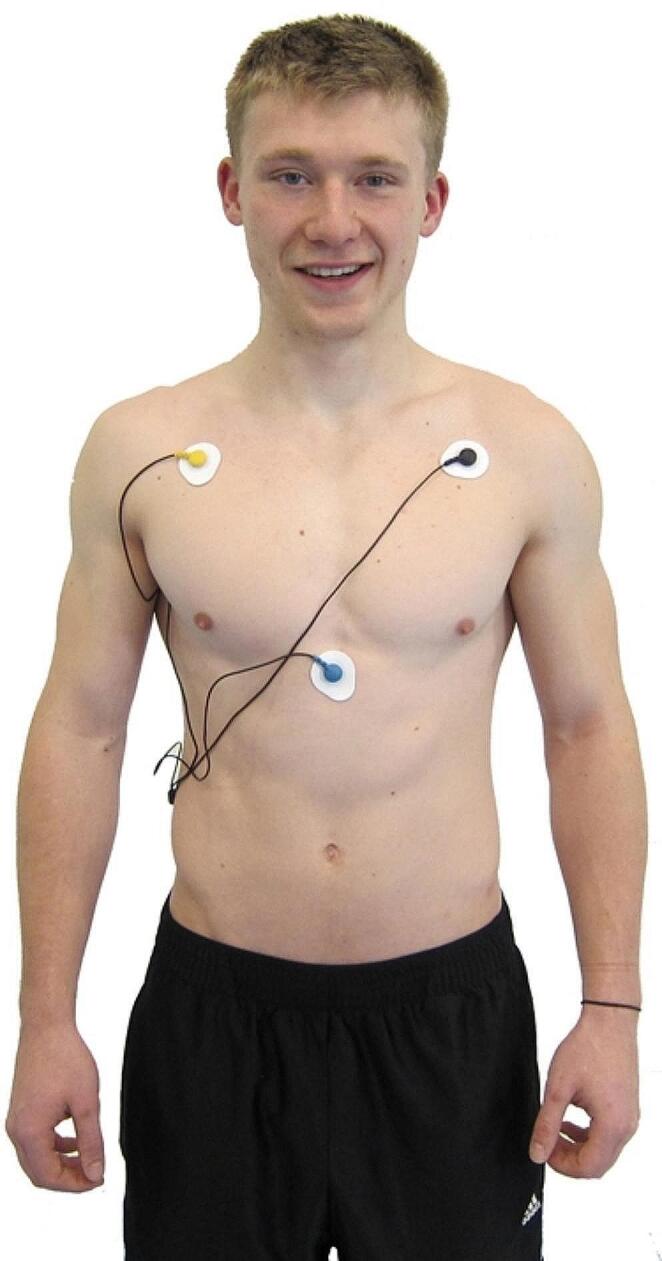
Active ECG electrodes on the upper torso

**Fig. 2:**
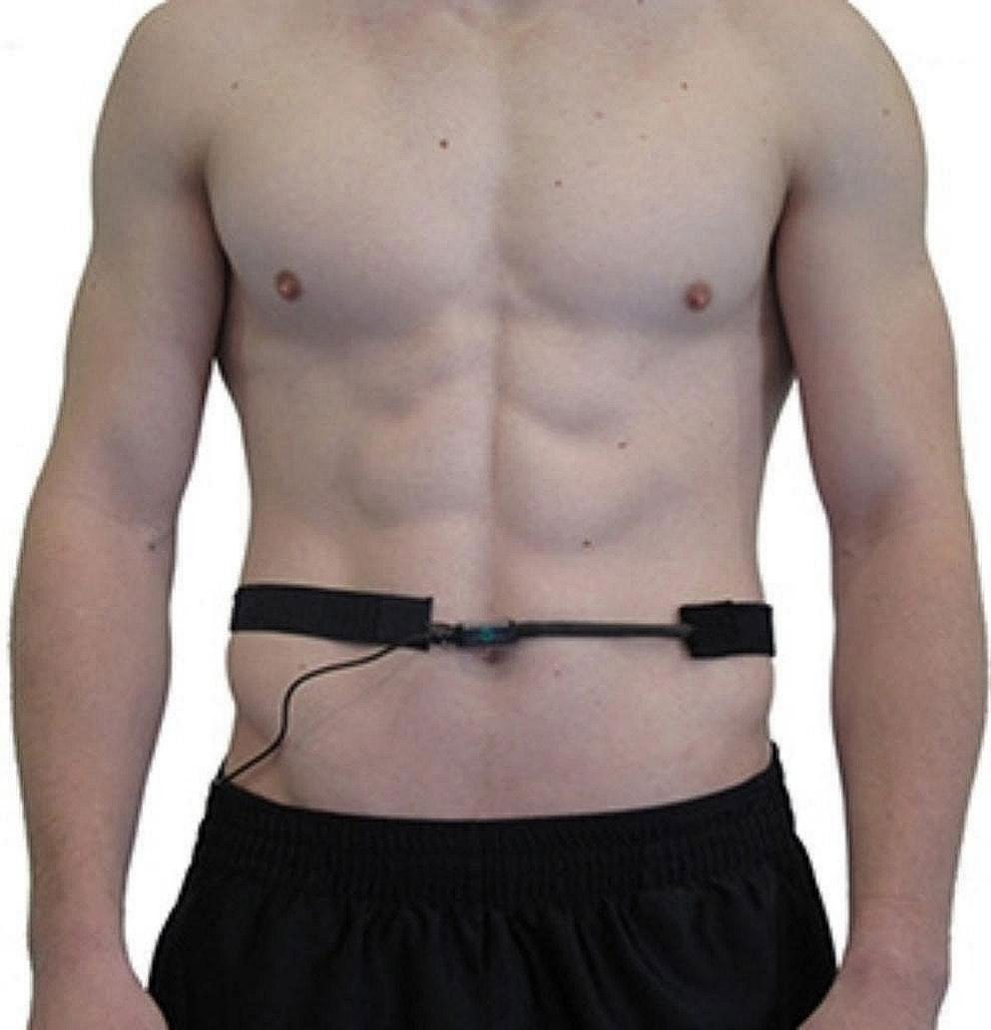
Respirometer placement

A Thought Technology ProComp Infiniti ™ system monitored ECG, HRV, and respiration. An EKG-Flex/Pro sensor measured heart rate (HR) and HRV from 0.05 Hz to 1 kHz at 2048 samples/s (s/s). We located active ECG electrodes on the upper torso (see Fig. 1). We positioned a Respiration-Flex/Pro respirometer over the navel (see Fig. 2) to measure abdominal excursion (e.g., the difference between the maximum and minimum expansion) and respiration rate (256 s/s). Participants sat upright in all conditions.

#### Dependent Variables

We measured HR, RR, the SDNN, RMSSD, and pNN50, and LF power. We did not report HF power since values obtained at 6-bpm would be invalid (Task Force, 1996). We manually artifacted data within CardioPro and detrended in Kubios using a smoothness priors procedure. Frequency-domain analysis utilized Welch’s periodogram (FFT) procedure. We used a natural log (Ln) transformation to yield a normalized distribution to satisfy the General Linear Model Repeated Measures better ANOVA’s assumptions.

#### Procedure

Before monitoring participants, we performed tracking tests on all channels. We randomly assigned participants to one of two IE ratio orders, 1:1 ➔ 1:2 or 1:2 ➔ 1:1, to minimize order effects. Each IE ratio condition lasted 5 min. We separated the IE ratio conditions with 3-min buffer periods to minimize carryover effects (Kazdin, 2016). We provided participants with 6-bpm animated pacing displays for 1:1 and 1:2 IE ratios. During each 5-min trial, researchers instructed participants to follow an animated ball.

#### Manipulation Check

Researchers visually confirmed compliance with respiration rate and IE ratio instructions by monitoring the respirometer waveform in real-time. Additionally, significance tests showed no difference in respiration rates between 1:1 and 1:2 IE ratio conditions, *t*(25) = -0.21, *p* = .83.

### Study Two

#### Participants

Sixteen undergraduates (8 women and 8 men), 18 to 22, participated in this study. They also completed one to two semesters of 6-bpm effortless breathing training. Researchers recruited participants from the Truman Center for Applied Psychophysiology. There were no participation incentives.

#### Apparatus

We used the Study 1 apparatus, placements, procedure, and manipulation checks.

#### Dependent Variables

We measured RR, HR, the SDNN, RMSSD, and pNN50, LF power, and sample entropy (SampEn). We did not measure HF power since values obtained at 6 bpm would be invalid. We used a natural log (Ln) transformation to yield a normalized distribution to satisfy the General Linear Model Repeated Measures better ANOVA’s assumptions. Table 2 lists the HRV variables monitored in this study.

**Table 2:** Descriptive statistics study one

	1:1 Ratio	1:2 Ratio
RR	6.02 (0.02)	6.03 (0.07)
HR	77.66 (12.76)	78.11 (13.01)
HR Max-Min	25.23 (10.91)	25.75 (11.16)
SDNN	100.69 (36.21)	101.82 (39.81)
RMSSD	67.90 (30.08)	69.76 (33.92)
pNN50	0.17 (0.10)	0.16 (0.12)
lnLF	7.66 (1.20)	7.71 (1.06)
SampEn	0.75 (0.17)	0.72 (0.18)

*Note* Tables include means with standard deviations in parentheses

**Table 3:** Descriptive statistics study two

	1:1 Ratio	1:2 Ratio
RR	6.01 (0.01)	6.04 (0.13)
HR	81.17 (11.53)	81.53 (12.61)
HR Max-Min	28.62 (9.49)	29.65 (9.77)
SDNN	98.98 (29.25)	94.02 (32.14)
RMSSD	67.31 (33.40)	65.33 (34.91)
pNN50	30.09 (16.72)	27.66 (16.35)
lnLF	4.51 (0.04)	4.49 (0.07)
SampEn	0.76 (0.16)	0.71 (0.16)

*Note* Tables include means with standard deviations in parentheses

#### Manipulation Check

Researchers visually confirmed compliance with respiration rate and IE ratio instructions by monitoring the respirometer waveform in real-time. Additionally, significance tests showed no difference in respiration rates between 1:1 and 1:2 IE ratio conditions, *t*(15) = -0.16, *p* = .88.

## Results

### Analytic Strategy

We completed a power analysis using G*Power 3.1.9.7 (Faul et al., 2007) to determine whether our sample size had sufficient power to detect an effect. We estimated the necessary sample size for the mean difference between two dependent variables in a within-subjects design where alpha is 0.05, power is 0.80, and Cohen’s *d* is approximately 1 based on prior research investigating IE ratios (Van Diest et al., 2014). The results suggest that a sample size of 10 participants is necessary to detect large effect sizes at 80% power.

We further tested whether the data for each experiment satisfied the assumptions for a dependent samples *t*-test. Specifically, we tested for both normality as well as outliers. We used the Shapiro-Wilk normality test to examine whether each dependent variable was normally distributed. We used Tukey’s method to detect and remove outliers (Tukey, 1977). We conducted dependent samples *t*-tests when dependent variables were normally distributed and Wilcoxon signed-rank test when dependent variables were not normally distributed. Finally, in the event of significant results, we reported both the original and adjusted *p*-value after applying a Bonferroni correction. This correction reduces the effect of inflated Type I error when conducting multiple tests (Bonferroni, 1936). Tables 1 and 2 provide descriptive statistics for both studies one and two.

### Study One

#### Respiration Rate

The Shapiro-Wilk normality test showed that RR distribution was normal for the 1:1 ratio measurement (*W* = 0.92, *p* = .07) but not normal for the 1:2 ratio measurement (*W* = 0.61, *p* < .001) after removing three outliers. Because the data were not normal for at least one of the paired measurements, we conducted a Wilcoxon signed ranks test, a nonparametric alternative to a dependent samples *t*-test. RR did not vary between the 1:1 (*M* = 6.02) and 1:2 ratio measurements (*M* = 6.03), Z(23) = -0.35, *p* = .73.

#### Heart Rate

The Shapiro-Wilk normality test showed that the HR distribution was normal for the 1:1 (*W* = 98, *p* = .80) and 1:2 ratio measurements (*W* = 0.97, *p* = .59). No outliers were detected or removed. Thus, we conducted a dependent samples *t*-test. HR did not change between the 1:1 (*M* = 77.66) and 1:2 ratio measurements (*M* = 78.11), *t*(25) = − 0.58, *p* = .57.

#### Heart Rate Max-Min

The Shapiro-Wilk normality test showed that the HR Max-Min distribution was normal for the 1:1 (*W* = 0.96, *p* = .38) and 1:2 ratio measurements (*W* = 0.97, *p* = .60). No outliers were detected or removed. Thus, we conducted a dependent samples *t*-test. HR Max-Min did not change between the 1:1 (*M* = 25.23) and 1:2 ratio measurements (*M* = 25.75), *t*(25) = − 0.43, *p* = .67.

#### SDNN

The Shapiro-Wilk normality test showed that the SDNN distribution was normal for the 1:1 (*W* = 0.98, *p* = .94) and 1:2 ratio measurements (*W* = 0.97, *p* = .67). No outliers were detected or removed. Thus, we conducted a dependent samples *t*-test. The SDNN did not change between the 1:1 (*M* = 100.69) and 1:2 ratio measurements (*M* = 101.82), *t*(25) = − 0.29, *p* = .77.

#### RMSSD

The Shapiro-Wilk normality test showed that the RMSSD distribution was normal for the 1:1 (*W* = 0.98, *p* = .95) and 1:2 ratio measurements (*W* = 0.95, *p* = .27) after removing one outlier. Thus, we conducted a dependent samples *t*-test. The RMSSD did not change between the 1:1 (*M* = 65.20) and 1:2 ratio measurements (*M* = 69.76), *t*(24) = -1.16, *p* = .26.

#### pNN50

The Shapiro-Wilk normality test showed that the pNN50 distribution was normal for the 1:1 measurement (*W* = 0.97, *p* = .50) but not in the 1:2 ratio (*W* = 0.92, *p* = .04). No outliers were detected or removed. Thus, we conducted the Wilcoxon signed-rank test. The pNN50 did not change between the 1:1 (*M* = 0.17) and 1:2 ratio measurements (*M* = 0.16), *Z*(26) = -0.22, *p* = .83.

#### Low-Frequency Power

The Shapiro-Wilk normality test showed that the LF power distribution was not normal for the 1:1 (*W* = 0.78, *p* < .001) or 1:2 ratio measurement (*W* = 0.83, *p* < .001) after removing two outliers. Thus, we conducted the Wilcoxon signed-rank test. LF power did not change between the 1:1 (*M* = 7.66) and 1:2 ratio measurements (*M* = 7.71), *Z*(24) = − 0.31, *p* = .75.

#### SampEn

The Shapiro-Wilk normality test showed that the SampEn distribution was normal for the 1:1 (*W* = 0.97, *p* = .62) and the 1:2 ratio measurement (*W* = 0.95, *p* = .32) after removing one outlier. Thus, we conducted the a dependent samples *t*-test. The SampEn did not change between the 1:1 (*M* = 0.75) and 1:2 ratio measurement (*M* = 0.72), *t*(23) = 1.33, *p* = .20.

### Study Two

#### Respiration Rate

After removing three outliers, the Shapiro-Wilk normality test showed that RR distribution was not normal for the 1:1 (*W* = 0.81, *p* = .01) or the 1:2 ratio measurement (*W* = 0.37, *p* < .001). Because the data were not normal for at least one of the paired measurements, we conducted a Wilcoxon signed ranks test, a nonparametric alternative to a dependent samples t-test. RR did not vary between the 1:1 (*M* = 6.01) and 1:2 ratio measurements (*M* = 6.04), Z(13) = -0.373, *p* = .47.

#### Heart Rate

The Shapiro-Wilk normality test showed that the HR distribution was normal for the 1:1 (*W* = 0.90, *p* = .09) but not the 1:2 ratio measurement (*W* = 0.82, *p* = .005). No outliers were detected or removed. Thus, we conducted a dependent samples *t*-test. HR did not change between the 1:1 (*M* = 77.66) and 1:2 ratio measurements (*M* = 78.11), *t*(25) = -0.58, *p* = .57.

#### Heart Rate Max-Min

The Shapiro-Wilk normality test showed that the HR Max-Min distribution was normal for the 1:1 (*W* = 0.97, *p* = .90) and 1:2 ratio measurements (*W* = 0.92, *p* = .20) after removing one outlier. Thus, we conducted a dependent samples *t*-test. HR Max-Min did not change between the 1:1 (*M* = 28.62) and the 1:2 ratio measurements (*M* = 29.83), *t*(14) = -0.89, *p* = .39.

#### SDNN

The Shapiro-Wilk normality test showed that the SDNN distribution was normal for the 1:1 (*W* = 0.97, *p* = .87) and 1:2 ratio measurements (*W* = 0.98, *p* = .97). Further, no outliers were detected or removed. Thus, we conducted a dependent samples *t*-test. The SDNN did not change between the 1:1 (*M* = 98.98) and 1:2 ratio measurements (*M* = 94.02), *t*(15) = 1.49, *p* = .16.

#### RMSSD

The Shapiro-Wilk normality test showed that the RMSSD distribution was normal for the 1:1 (*W* = 0.95, *p* = .53) and 1:2 ratio measurements (*W* = 0.93, *p* = .21). No outliers were detected or removed. Thus, we conducted a dependent samples *t*-test. The RMSSD did not change between the 1:1 (*M* = 67.31) and 1:2 ratio measurements (*M* = 65.33), *t*(15) = 0.46, *p* = .65.

#### pNN50

The Shapiro-Wilk normality test showed that the pNN50 distribution was normal for the 1:1 (*W* = 0.97, *p* = .82) and 1:2 ratio measurements (*W* = 0.95, *p* = .49). No outliers were detected or removed. Thus, we conducted a dependent samples *t*-test. The pNN50 did not change between the 1:1 (*M* = 30.09) and 1:2 ratio measurements (*M* = 27.66), *t*(15) = -1.55, *p* = .14.

Low-Frequency Power.

The Shapiro-Wilk normality test showed that the LF power distribution was normal for the 1:1 (*W* = 0.95, *p* = .62) and 1:2 ratio measurements (*W* = 0.91, *p* = .17) after removing three outliers. Thus, we conducted a dependent samples *t*-test. LF power did not change between the 1:1 (*M* = 4.51) and 1:2 ratio measurements (*M* = 4.50), *t*(12) = 0.69, *p* = .51.

#### SampEn

The Shapiro-Wilk normality test showed that the SampEn distribution was normal for the 1:1 (*W* = 0.96, *p* = .62) and 1:2 ratio measurements (*W* = 0.97, *p* = .89). No outliers were detected or removed. Thus, we conducted a dependent samples *t*-test. SampEn did not change between the 1:1 (*M* = 0.76) and the 1:2 ratio measurements (*M* = 0.71), *t*(15) = 1.45, *p* = .17.

## Discussion

The current manuscript examined whether a 1:1 or 1:2 IE ratio produces different HRV time-domain, frequency-domain, or nonlinear metrics when participants breathe within the RF range at 6 bpm. Strengths of this study included (1) examining the literature for common methodological challenges, (2) designing protocols that eliminate or mitigate these challenges, and (3) conducting two experiments to test the replicability of findings. Across an original experiment and a replication, participants experienced no difference in HRV time-domain, frequency-domain, or nonlinear metrics when breathing at a 1:1 or 1:2 ratio within the RF range. Thus, these findings suggest that breathing at a 1:2 IE ratio may not alter valued HRV metrics when breathing in the RF range.

We used the Cochrane risk-of-bias tool (Higgins et al., 2011) to outline common methodological challenges observed in each reviewed study, including the current study. The most common sources of bias involved the randomization process, intervention delivery, and the selection of reported results. Specifically, four studies neglected to randomize their participants and/or counterbalance the conditions (Bae et al., 2021; Klintworth et al., 2012; Strauss-Blasche et al., 2000; Van Diest et al., 2014). Additionally, eight studies did not confirm that the participants breathed at their assigned RR or IE ratios (Bae et al., 2021; Cappo & Holmes, 1984; Edmonds et al., 2009; Klintworth et al., 2012; Laborde et al., 2021; Lin et al., 2014; Strauss-Blasche et al., 2000; Van Diest et al., 2014). Finally, three studies reported only significant findings for a select group of the metrics evaluated (Bae et al., 2021; Cappo & Holmes, 1984; Paprika et al., 2014), thereby increasing the possibility of inflated Type I error rates (Kepes et al., 2014). In sum, only the current study met the methodological challenges and tested IE ratio effects at 6 bpm.

Following a review of methodological challenges, we outline four strategies to limit bias when investigating IE ratios. First, participants must breathe at the assigned RR, and researchers should confirm these patterns using statistical analyses when possible. Given that RR affects HRV metrics (Lehrer & Gevirtz, 2014), it represents a common and potent confound that limits the validity of findings. Second, participants must breathe at the assigned IE ratios, and researchers should confirm these patterns with real-time participant feedback. For example, researchers should monitor and actively encourage participants when their breathing matches the assigned IE ratio and offer constructive feedback when they notice discrepancies (e.g., breathing at the wrong ratio). We also advise that researchers rely on pacing cues (e.g., visual, auditory), when possible, as these offer participants more immediate guidance regarding breathing patterns than other methods (e.g., verbal instruction). We recognize that metrics that estimate the degree to which the achieved respiration waveform matches the pacing display are not widely available. As such, we urge the developers of data acquisition systems to add functions that calculate these values. Third, we recommend that researchers avoid confounding order effects by counterbalancing conditions using an evidence-based method (e.g., Latin squares, randomization). Finally, we recommend that researchers report null results with as much detail as the significant findings. Failure to report null findings has downstream consequences for the accuracy of meta-analytic reviews (Kepes et al., 2014), as publication bias artificially inflates Type I error.

## Limitations

Our studies had important limitations that should provide directions for future research. First, we sampled a population of undergraduate students, which is common in psychological research but often limits the generalizability of findings (Henrich et al., 2010). Further investigations should recruit participants who better resemble clinical populations in age and health to increase external validity. Second, we trained participants to breathe at 6 bpm rather than their individual RF. Because individuals maximize RSA by breathing at their unique RF (Lehrer & Gevirtz, 2014), future work should individualize the breaths per minute to suit their participants. Third, we examined only two IE ratios. Future work should include a greater variety, such as the studies in this review, as this variety will help detect boundary effects. Fourth, data acquisition software should measure the proportion of time participants breathing waveforms matched the pacing display, thereby confirming their adherence to the IE ratio. Finally, participants in both study one and study two were trained over the 5–6 weeks to breathe at a 1:1 IE ratio, then tested on the effects of breathing at a 1:1 or 1:2 IE ratio. We recommend that future work should compare the longitudinal effects of training participants to breathe at either a 1:1 or 1:2 IE ratio.

## Clinical Implications

The current studies found no differences between the 1:1 and 1:2 IE ratios on HRV metrics. Thus, clinicians should simplify RF assessment using a 1:1 ratio, provided their client’s preference (Fisher & Lehrer, 2022). Likewise, clinicians should consider SPB at 1:1 if it increases client success, but only if this change does not compromise breathing health (e.g., over breathing).
